# Jiyuan Oridonin A Overcomes Differentiation Blockade in Acute Myeloid Leukemia Cells With MLL Rearrangements *via* Multiple Signaling Pathways

**DOI:** 10.3389/fonc.2021.659720

**Published:** 2021-03-26

**Authors:** Mei Qu, Yu Duan, Min Zhao, Zhanju Wang, Mengjie Zhao, Yao Zhao, Haihua Wang, Yu Ke, Ying Liu, Hong-Min Liu, Liuya Wei, Zhenbo Hu

**Affiliations:** ^1^ Laboratory for Stem Cell and Regenerative Medicine, Affiliated Hospital of Weifang Medical University, Weifang, China; ^2^ School of Pharmacy, Weifang Medical University, Weifang, China; ^3^ School of Pharmacy, Zhengzhou University, Zhengzhou, China

**Keywords:** differentiation therapy, acute myeloid leukemia with MLL gene rearrangements, Jiyuan oridonin A, martens tretinoin response up pathway, hematopoietic cell lineage pathway, cell adhesion pathway

## Abstract

Differentiation therapy with all-trans-retinoic acid (ATRA) in acute promyelocytic leukemia (APL), a subtype of acute myeloid leukemia (AML), has been extremely successful in inducing clinical remission in APL patients. However, the differentiation therapy of ATRA-based treatment has not been effective in other subtypes of AML. In this study, we evaluated a small molecule of *ent*-kaurene diterpenoid, Jiyuan oridonin A (JOA), on the differentiation blockade in AML cells with the mixed lineage leukemia (MLL) gene rearrangements (MLLr) in MV4-11, MOLM-13 and THP-1 cells. We found that JOA could significantly inhibit the proliferation of MOLM-13, MV4-11 and THP-1 cells. Moreover, JOA promoted cell differentiation coupled with cell-cycle exit at G0/G1 and inhibited the colony- forming capacity of these cells. We showed that the anti-proliferative effect of JOA attributed to cell differentiation is most likely through the martens tretinoin response up pathway in the MOLM-13 cell line, and the hematopoietic cell lineage pathway by the inhibition of c-KIT expression and cell adhesion pathway in the THP-1 cell line. Our findings suggest that JOA could be a novel therapeutic agent against human MLLr acute myeloid leukemia.

## Introduction

Acute myeloid leukemia (AML) is the most common acute leukemia in adults and the second most common form of acute leukemia in children. AML is a malignancy of hematopoietic stem cell, with distinctive features of excessive proliferation and impaired differentiation (1, 2). The biology of AML cells is complex and involves multiple interdependent molecular pathways. The traditional chemotherapies are the mainstay of treatment for the past 50 years. However, a 5-year overall survival (OS) for patients younger than 60 years is about 40%. For those older than 60 years, the 5-year OS of only 10~20% has been achieved (3). In AML, the normal differentiation pathway is impaired and the cells persistently proliferate without undergoing terminal apoptosis (4, 5). If the leukemic cells could be forced to stop proliferating and turned to differentiation, the malignancy would be controlled. Hence, the idea toward eliminating the maturation block and inducing differentiation contributes to the development of the differentiation therapy (6, 7). Differentiation therapy is easily monitored because hematopoietic cells have a broad range of surface biomarkers and specific morphological features (8). The differentiation-inducing agent, All-trans retinoic acid (ATRA, tretinoin) is effective in the treatment of acute promyelocytic leukemia (APL), the M3 subtype of AML (9). However, there has been no remarkable progress in the treatment of the other 90% of AML cases. Moreover, AML with the mixed lineage leukemia (MLL) gene rearrangements (MLLr) found in about 10% of AML often associated with subtypes M4 and M5 of AML, that are associated with poor prognosis (10, 11). Therefore, finding new differentiation inducers against MLLr of AML is urgently needed. Natural products have been shown to possess multi-faceted mechanism that can target multiple pathways that are de-regulated in cancer cells to achieve greater therapeutic efficacy.

Our previous work had shown that OGP46 have been shown to have anti-proliferative effect in leukemic cells such as BaF3-T315I and K562 cells (12). OGP46 also possessed anti-proliferative activity in solid tumor cells including SMMC-7721.25, A549, Eca-109, and MCF-7 cells (13–15). Jiyuan oridonin A (JOA), a kaurene diterpenoid compound isolated from Isodon rubescensin, has similar structure to OGP46. In the current study, we explored whether JOA possesses anti-leukemia activity against MLLr AML cells including MV4-11, MOLM-13 and THP-1.

## Materials and Methods

### Chemicals

**Figure 1:**
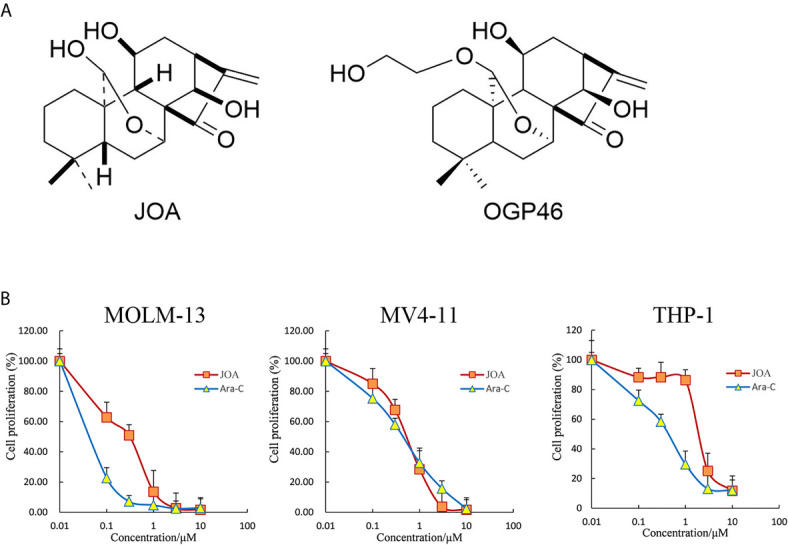
JOA reduces the proliferation of MV4-11, MOLM-13 and THP-1 cells. **(A)** The chemical structure of JOA and OGP46. **(B)** The effect of JOA in inhibiting cellular proliferation. Cells were treated with different concentrations of JOA or Ara-C (0- 10 μM) for 72 h, and subjected to CCK-8 assay. Error bars represent the mean ± SD. The CCK-8 assay was carried out for four times in triplicate.

We have prepared JOA with purity of 98.5% by isolating it from Isodon rubescensin (15), which is similar to OGP46. The sole difference of the two compounds is the substituent of the C20. The chemical structure of JOA with a molecular weight of 348.2 and OGP46 were shown in **Figure 1A**. JOA and Cytosine arabinoside (Ara-C) were dissolved in dimethyl sulfoxide (DMSO) to 10 mM stock solutions and stored at -20°C. The same concentration of DMSO as corresponding to JOA solution diluted in RPMI 1640 medium was used as the control. The final DMSO concentrations during all incubations were not more than 0.1%, which had no observable toxic effects to cells. Fluorescein Isothiocyanate (FITC)/Annexin V Apoptosis Detection Kit and Propidium iodide (PI)/RNase staining solution were obtained from BD Biosciences (San Jose, CA, USA). Cell Counting Kit-8 (CCK-8) was purchased from Solarbio (Beijing, China). PE anti- CD13 (cat #301704 RRID: AB_314180), FITC anti- CD14 (cat #301804, RRID: AB_314186) and PE anti- CD15 (cat #301906, RRID: AB_314198) were obtained from Biolegend (Inc, San Diego, CA, USA). MethoCult H4100 (cat #04100) and H4435 (cat #04435) were obtained from STEMCELL Technologies Inc. (Vancouver, BC, Canada). Antibodies against GAPDH (Cat #5174) was obtained from Cell Signaling Technology (Beverly, MA, USA). Antibodies against ALDH3A1 (EPR7406) and c-KIT (EPR22566-344) were obtained from Abcam (Cambridge, MA, USA). Antibody against CALML6 (SAB 1402033) was purchased from Sigma-Aldrich. The 2×SYBR Green qPCR Mix (cat #AH0104-B), SPARKscript II RT Plus kit (cat #AG0304-B), Spark ECL Plus A (cat #ED0015-C), Spark ECL Plus B (cat #ED0016-C) and RIPA buffer (SparkJade EA0002) were purchased from SPARKJADE (Shandong, China).

### Cell Lines and Cell Culture

MOLM-13 (AML with MLLr expressing FLT3/ITD mutation, DSMZ No.: ACC 554), MV4-11 (AML with MLLr expressing FLT3/ITD mutation, DSMZ No.: ACC 102), and THP-1 (AML with MLLr expressing wild -type FLT3, DSMZ No.: ACC 16) cell lines were used. These cell lines were grown in RPMI-1640 medium containing 10% FBS and streptomycin/penicillin (1%) with 5% CO_2_ at 37°C.

### Cell Proliferation Assay

The proliferation of MV4-11, MOLM-13, and THP-1 cells was evaluated by CCK-8 assay. Briefly, cells were seeded in 96-well plate with about 5×10^3^ cells in each well. The cells were treated with JOA or Ara-C (0- 10 μM) after 24 h of incubation. 72 h later, CCK-8 reagent (10 μL) was added to each well, and cells were incubated at 37°C for 4 h. The light absorbance at 450 nm was measured by using an Opsys microplate reader (Dynex Technologirs, Chantilly, VA, USA). Results are expressed as percent of cell viability normalized to DMSO-treated control cells.

### Colony Formation Assay

MV4-11, MOLM-13 and THP-1 cells were cultured with JOA (0- 2 μM) in 2.6% methycellulose medium containing 10% FBS in a 24- well flat-bottomed plate for 12 days. The number of individual colonies consisting of more than approximately 50 cells was counted by a CX43 microscope (Olympus, Shinjuku-ku, Tokyo, Japan) with an Olympus EP50 camera (Olympus, Shinjuku-ku, Tokyo, Japan).

### Cell Cycle Analysis

MV4-11, MOLM-13 and THP-1 cells were incubated with JOA for 48 or 72 h. After treatment, cells were collected and fixed with 70% pre-cold ethanol in PBS and stored at -20°C for at least 24 h. Then the cells were stained with 50 mg/mL PI and 100 mg/mL RNase A for 30 min in the dark at room temperature. Finally, flow cytometry was used to detect the percentage of cells in the sub-G1, G0/G1, S, and G2/M phases with a Beckman Coulter DxFLEX flow cytometer (Florida, Miami, USA). The data was analyzed and fitted by ModFit software.

#### Cell Apoptosis Analysis

MV4-11, MOLM-13 and THP-1 cells were treated with JOA (0.25- 4 μM) or Ara-C (0.5- 4 μM) for 72 h, collected and resuspended in 1× binding buffer. Cells were incubated with FITC Annexin V and PI double labeling for 30 min in the dark at room temperature and measured by flow cytometry.

#### Analysis of Cell Morphology

MV4-11, MOLM-13 and THP-1 cells were incubated with JOA or Ara-C for 72 h and then collected. Slides were made by cytospin and subsequently air dried. The cells were stained with Wright-Giemsa and observed for morphological features using a light microscope.

#### Cell Surface Antigens Measurement

After treatment with JOA for 72 h, the cells were centrifuged, collected and washed, and incubated with different antibodies for 30 min at room temperature in the dark. Cell surface antigens analysis was carried out by using a flow cytometer.

#### mRNA-Sequencing Analysis

mRNA-Sequencing (mRNA-seq) was performed for MOLM-13 and THP-1 cells. As described in a previous work [12], after incubating with JOA for 48 h, cells were collected for RNA extraction. Sequencing libraries were prepared and then sequenced using 150 bp pair-end strategies with an Illumina HiSeq X10 instrument (Annoroad Genomics, Beijing, China). The mRNAs levels were estimated using FPKM (fragments per kilobase of exon per million fragments mapped). Differential expression analysis was performed using DESeq R packages. A corrected p value of 0.05 and absolute value of log 2 FC (fold change) ≥ 0.58 was set as a threshold of differential expression of genes (DEGs). Gene Set Enrichment Analysis (GSEA) was conducted and C2 curated functional gene sets from the Molecular Signature Database (http://www.gsea-msigdb.org/gsea/msigdb) for MOLM-13 cell line. In addition, the method of geometric test was used to enrich the DEGs from the Gene Ontology and Kyoto Encyclopedia of Genes and Genomes (KEGG) databases in THP-1 cell line. The clusterProfiler package of R software was used to tthe enrichment analysis of DEGs in both methods. The pathways with an adjusted p value of < 0.05 were considered significantly enriched.

### Verification of Expression Difference Genes by Real-time PCR (RT-PCR)

The total RNAs of MOLM-13 and THP-1 cells treated with JOA were extracted using the Trizol reagent. Reverse transcription for first strand of cDNA was performed using the Primerscript RT reagent kit with gDNA Eraser. The following primers were used to amplify cDNA: forward GAPDH 5’-TGGGTGTGAACCATGAGAAGT-3’, reverse GAPDH 5’-TGAGTCCTTCCACGATACCAA-3’; forward c-KIT 5’-TGACTTACGACAGGCTCGTG-3’, reverse c-KIT 5’-CCACTGGCAGTACAGAAGCA-3’; forward ALDH3A1 5’-TGTGTCAAAGGCGCCATGAGCAAG-3’, reverse ALDH3A1 5’-GGCGTTCCATTCATTCTTGTGCAG-3’; forward CALML6 5’-GGGCTACATTGACTGG AACACAC-3’, reverse CALML6 5’-CCTCATAGTCGATGGTCCTGTC-3’.

#### Western Blotting Analysis

After treatment with JOA for 72 h, the MOLM-13 and THP-1 cells were lysed by RIPA buffer with proteinase inhibitors. The protein lysates were separated by sodium dodecyl sulfate polyacrylamide gel (SDS-PAGE), then transferred to PVDF membrane. After blocked by 10% skimmed milk, the membranes were incubated with specific primary antibodies overnight at 4°C, followed by the incubation with goat anti‐rabbit or goat anti‐mouse immunoglobulin G (IgG) antibodies for 1 hour at room temperature. The expression of proteins was visualized using an enhanced chemiluminescence (ECL) reagent detection system (Amersham Imager 600; GE Healthcare Biosciences, Pittsburgh, PA, USA).

### Statistical Analysis

All experiments were repeated at least three times unless otherwise stated. The data were represented as mean ± SD. Statistical analysis were performed with Student’s t test for two-group comparisons and using one-way ANOVA with Tukey’s *post hoc* test for multigroup comparisons. and p < 0.05 or p < 0.01 were considered statistically significant.

## Results

### JOA Possesses Anti-Leukemic Activity Against MLLr AML Cell Lines

Cells were treated with different concentrations of JOA or Ara-C (0- 10 μM) for 72 h, and CCK-8 assay was carried out to test the effect of JOA on cell proliferation as compared to Ara-C. As shown in **Figure 1B**, JOA significantly reduced the proliferation of MV4-11, MOLM-13 and THP-1 cells with the IC_50_ value of 0.62, 0.42, 2.19 μM, respectively, that were comparable with those of Ara-C which was 0.52, 0.07 and 0.50 μM, respectively. This finding revealed that JOA has the activity against the proliferation of MLLr AML cells. Moreover, JOA has similar anti- proliferative effect as Ara-C on MV4-11 cells. In addition, MOLM-13 and MV4-11 cells, that carried FLT3/ITD mutation, are more sensitive to JOA than THP-1 cells that had wild -type FLT3.

### JOA Inhibits Colony Formation of MLLr AML Cells

**Figure 2:**
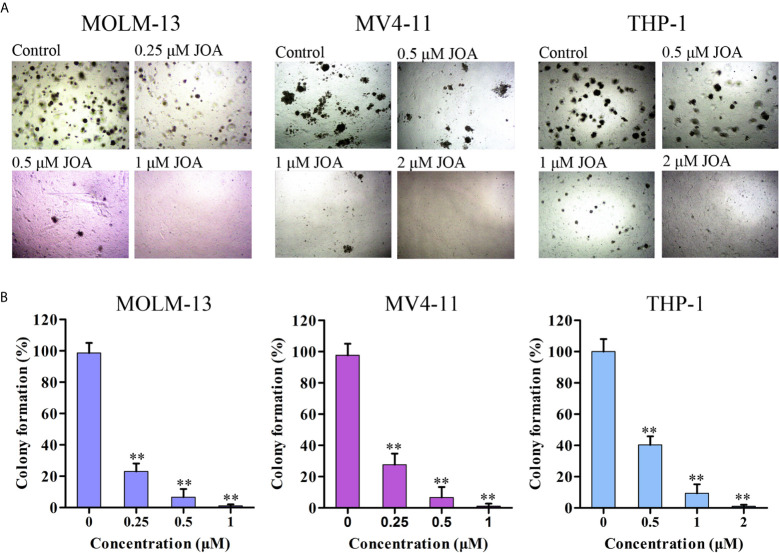
JOA suppresses the colony formation in MV4-11, MOLM-13 and THP-1 cells. **(A)** Cells were treated with JOA at the concentrations of 0.25- 2 μM for 12 days then the cell morphology was observed under light microscopy. **(B)** Graph bars show the number of colonies (**p < 0.01). The colony formation assay was performed for three times.

The effect of JOA on the colony forming capacity of MV4-11, MOLM-13 and THP-1 cells was next investigated. Cells were treated with JOA at the concentrations of 0.25- 2 μM for 12 days and the formation of colonies was observed under a microscope. It was found that JOA significantly decreased the number of colonies in a concentration-dependent manner. Moreover, the MV4-11, MOLM-13 and THP-1 cells failed to form colonies when treated with 1-2 μM JOA (**Figure 2**). This result indicated that the colony- forming capacity of MLLr AML cells was significantly inhibited by JOA treatment.

### JOA Induces Cell Cycle Arrest at G0/G1 in MLLr AML Cell Lines

**Figure 3:**
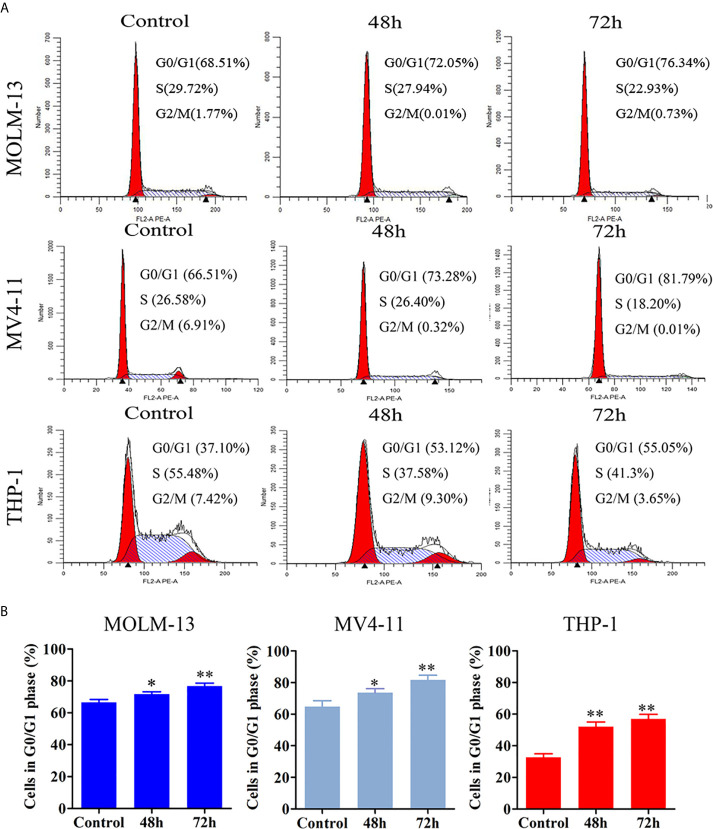
JOA induces cell cycle exit in MV4-11, MOLM-13 and THP-1 cells. **(A)** MV4-11, MOLM-13 and THP-1 cells were treated with 0.5, 0.5 or 2 μM JOA, respectively for 48 h or 72 h, then flow cytometry was used to detect the percentage of cells in various phase of the cell cycle. **(B)** Graph bars show the percentage of cells in G0/G1 phase. (*p < 0.05, **p < 0.01). The cell cycle analysis was performed for four times.

The effect of JOA on cell-cycle progression was evaluated in MOLM-13, MV4-11 and THP-1 cells. MV4-11, Cells were treated with 0.5, 0.5, 1 or 2 μM JOA, respectively for 48 h or 72 h, and the percentage of cells in various phase of cell cycle was analyzed with flow cytometry. As shown in **Figure 3**, the percentage of cells in G0/G1 phase increased in a time-dependent manner in these cells. This result suggested that JOA inhibits cell proliferation through inducing a G0/G1 cell cycle exit in MLLr AML cells.

### JOA Induces Minimal Signs of Apoptosis in MLLr AML Cell Lines

**Figure 4:**
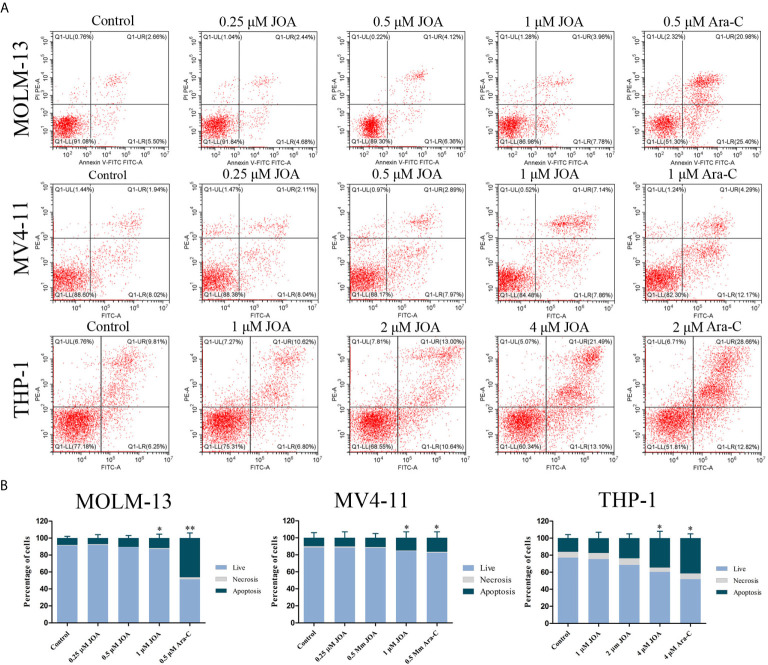
JOA does not induce significant apoptosis in MV4-11, MOLM-13 and THP-1 cells. **(A)** Cells were treated with JOA or Ara-C for 72 h and cell apoptosis was determined by flow cytometric analysis. **(B)** Graph bars show the percentage of living cells and cells undergoing necrosis/apoptosis. MOLM-13, MV4-11 cells were treated with 0.25, 0.5, 1 μM JOA or Ara-C (0.5 or 1 μM) and THP-1 were treated with 1, 2, 4 μM JOA or 4 μM Ara-c for 72h. (*p < 0.05, **p < 0.01). The cell apoptosis analysis was performed for four times.

In order to examine whether the anti-proliferation of JOA is associated with the induction of apoptosis, MV4-11, MOLM-13, and THP-1 cells were treated with different concentrations of JOA or Ara-C for 72 h and cell apoptosis was determined by flow cytometric analysis. As shown in **Figures 4A**, **B**, no significant apoptosis was observed when MOLM-13 and MV4-11 cells were incubated with JOA (0.25, 0.5, or 1 μM). Similarly, minimal signs of apoptosis were found in THP-1 cells treated with 1 or 2 μM JOA. In contrast, Ara-C led to the obvious apoptosis in all three cell lines at indicated concentration. This data revealed that the cell-cycle exit was not associated with cell apoptosis in MV4-11, MOLM-13 and THP-1 cells treated with JOA at 0.5, 0.5, 2 μM, respectively. The concentration of JOA will be used to treat the cell lines in subsequent experiment.

### JOA Promotes Cell Differentiation in MLLr AML Cell Lines

**Figure 5:**
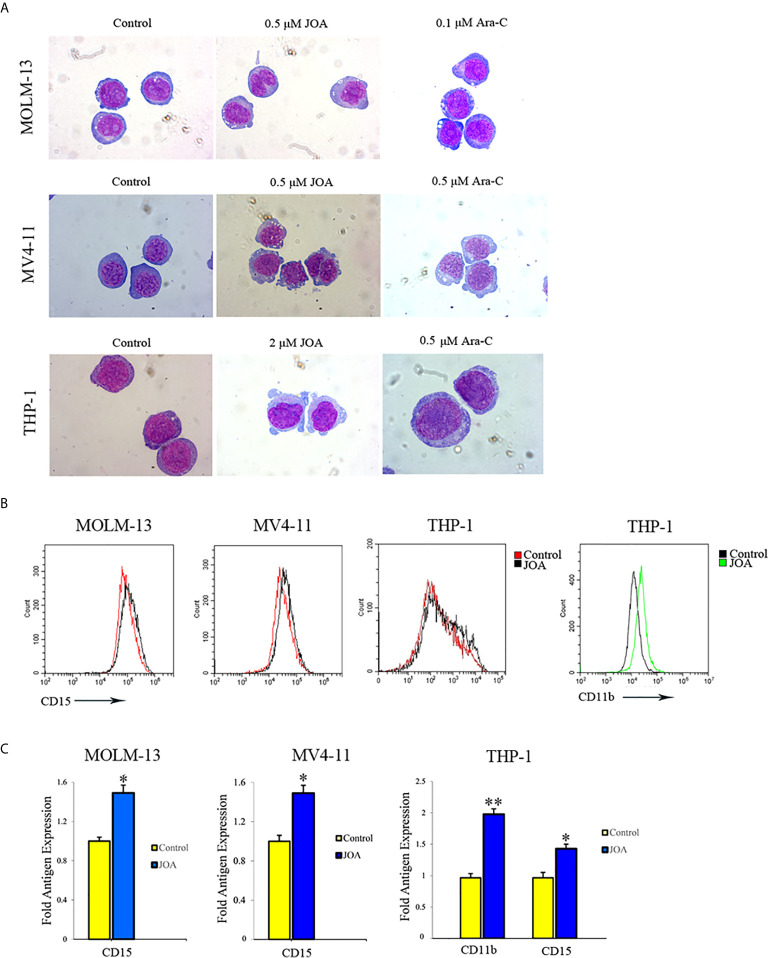
JOA induces differentiation of MV4-11, MOLM-13 and THP-1 cells. **(A)** Wright-Giemsa staining images of cells captured by oil immersion lens (×1,000). **(B)** The expression of cell-surface antigens was measured by flow cytometry. **(C)** Graph bars show the mean fluorescence intensity (MFI) of antigens. MV4-11, MOLM-13 and THP-1 cells were incubated with 0.5, 0.5 or 2 μM JOA, or Ara-C at the concentration of 0.1, 0.5 or 0.5 μM for 72 h, respectively (*p < 0.05, **p < 0.01). The figures are representative of three independent experiments.

Because JOA did not cause the apoptosis of MV4-11, MOLM-13 and THP-1 cells at the indicated concentration, morphology and flow cytometry analysis were carried out to assess the differentiation of these cells treated with JOA. A set of four myeloid biomarkers (CD11b, CD13, CD14 and CD15) were assessed to analyze the cell phenotype. It was seen that all the three cell lines showed increased cell size with a decrease in the nuclear-cytoplasmic ratio, indicating that JOA induced the cell differentiation accompanied with morphological changes, while Ara-C did not promote cell differentiation (**Figure 5A**). Moreover, JOA increased the expression of CD15 (a monocyte/macrophage biomarker) in all these three leukemia cell lines (**Figures 5B, C**). In addition, JOA significantly increased the expression of CD11b (ITGAM, a monocyte/granulocyte biomarker) in THP-1 cells. These results suggested that JOA induces cell differentiation in MLLr AML cells.

### JOA Induces Cell Differentiation Is Associated With Multiple Signaling Pathways Depending on Cell Type

**Figure 6:**
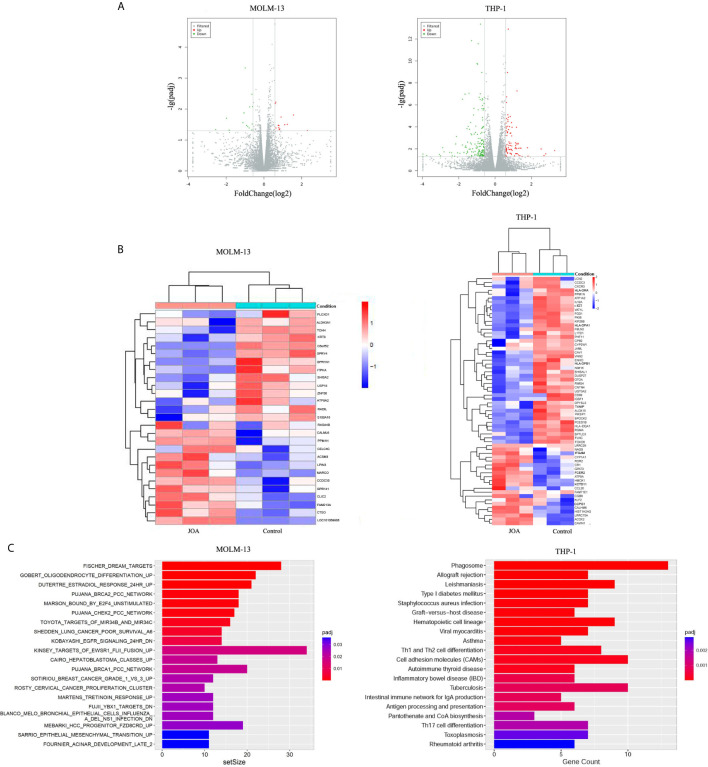
JOA promotes cell differentiation *via* multiple pathways depending on cell type. MOLM-13 and THP-1 Cells were incubated with 0.5 or 2 μM JOA, respectively for 72 h. **(A)** Volcano plots of MOLM-13 and THP-1 cells. **(B)** The heatmap of DEGs. The heatmap bars from blue to red represents the expression levels of genes from lower to higher. (100 genes with p < 0.05 and |log 2 FC| >0.58 based on their p value in the THP-1 cells). **(C)** KEGG pathway analyses on the differentially expressed genes. MOLM-13 and THP-1 cells were incubated with 0.5, or 2 μM JOA for 72 h, respectively. The figures are representative of two independent experiments.

To elucidate the molecular mechanism of cell differentiation- mediated by JOA, we performed global gene expression analyses by mRNA-seq in MOLM-13 and THP-1 cells. The volcano plots of MOLM-13 and THP-1 cells were shown in **Figure 6A**. A total of 14 genes were down-regulated and 13 genes were up-regulated in MOLM-13 cells. Similarly, the expression of 165 genes decreased, and 83 genes increased in THP-1 cells, indicating that the different effect of JOA on the expression of different genes. This result suggested that JOA does not universally affect the mRNA expression of all genes in these cells. As shown in **Figure 6B**, aldehyde dehydrogenase 3A1 (ALDH3A1) was markedly down-regulated, whereas the calcium binding protein calmodulin (CALML6) and LPIN3 were significantly up-regulated in MOLM-13 cells incubated with JOA. Simultaneously, genes such as ITGAM (CD11b), CR1, FCER2, OCLN, HMOX1 were significantly up-regulated and genes including c-KIT, cell adhesion molecules (CD99, HLA-DQA1, HLA-DRA, HLA-DPA1, HLA-DPB1) were significantly down-regulated in THP-1 cells after JOA treatment. GSEA analysis showed that the most optimal pathway induced by JOA was martens tretinoin response up (AML-induced differentiation) pathway in MOLM-13 cells. In contrast, the DEGs were enriched in the hematopoietic cell lineage, cell adhesion molecules and phagosome pathways in THP-1 cells (**Figure 6C**).

**Figure 7:**
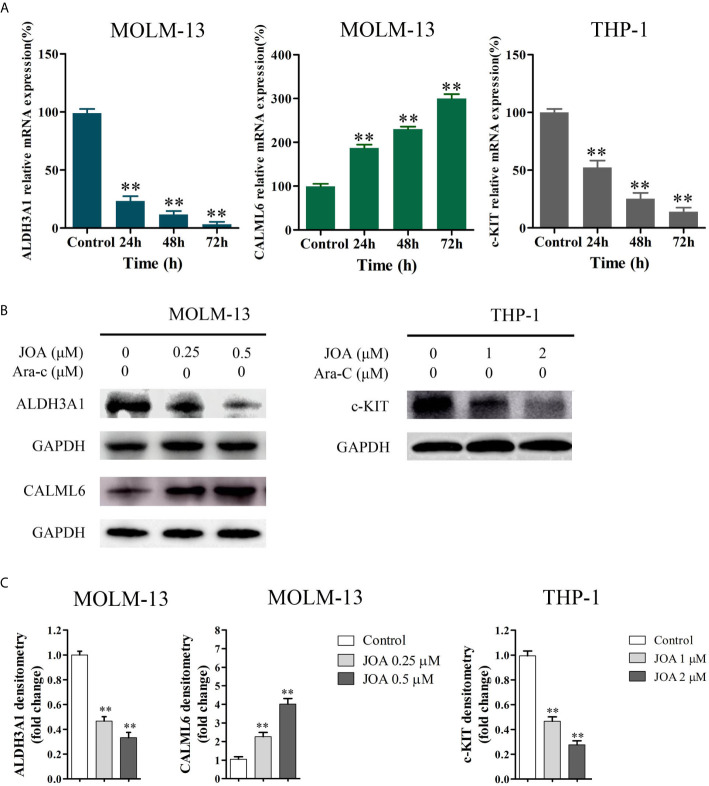
The RT-PCR and Western blotting analysis of DEGs in MOLM-13 and THP-1 cells. **(A)** The effects of JOA on the mRNA expression of ALDH3A1, CALML6 and c-KIT evaluated by RT-PCR **(B)** ALDH3A1, CALML6 and c-KIT protein levels measured by Western blotting analysis. **(C)** Graph bars show the protein expression visualized and quantified by AI600 imager. MOLM-13 and THP-1 cells were incubated with 0.5, or 2 μM JOA for 72 h, respectively. (**p < 0.01). The figures are representative of four independent experiments.

The DEGs identified by mRNA-seq paly important role in cell differentiation. To confirm their expression in the MLLr AML cells, RT- PCR and Western blotting were carried out in MOLM-13 and THP-1 cell lines. It was found that JOA treatment markedly changed the expression of mRNA and protein of ALDH3A1 and CALML6 in MOLM-13 cells and the expression of c-KIT was significantly down-regulated at both transcription and protein level in THP-1 cells (**Figure 7**). These results were in consistent with the results of mRNA-seq.

## Discussion

AML accounting for 75% of acute leukemia, is a heterogeneous disease characterized by accumulation of immature myeloid cells in bone marrow and peripheral blood with abnormal proliferation and differentiation blockade (9, 16). In the past decades, chemotherapy consisting of Ara-C and anthracycline is used for the first-line treatment of AML (17). Although the complete remission rate is high initially, the adult five-year overall survival of AML patients is less than 40% because of chemotherapy resistance (18). Recently, the recognition of origins and drivers of the pathogenesis of AML enabled the development of novel therapeutics in targeting multiple signaling pathways such as proteasome pathway, apoptosis, c-Myc signaling and hedgehog pathway (19, 20). The differentiation therapy using ATRA dramatically improved the long-term clinical outcome for APL patients, which attributed to the ATRA-induced degradation of PML/RARα by the proteasome pathway. Moreover, ATRA treatment converted the AML subtype from a bad prognostic leukemia to a curable one (21). However, clinical effectiveness of ATRA-based differentiation therapy has no effect in other AML subtypes (22). In addition, MLL-rearranged AML has been associated with poor outcome despite intensive chemotherapy. Therefore, the development of potent small molecules to overcome these drawbacks may be offer alternative or complementary therapies. In the current study, we investigate the role of a novel diterpenoid compound JOA on the differentiation of MLLr AML cells.

The analog of JOA, OGP46, have been found to exert antileukemic effect on various CML cell lines, such as K562 and BaF3 cells that expressed BCR-ABL wild-type or mutations, by cell differentiation (12). Here, we found that JOA significantly inhibited cell proliferation, colony formation by cell differentiation of MLLr AML cells, as evidenced by the morphological changes, increasing expression of the cell surface antigen CD15 or CD11b. We found that the cell differentiation was coupled with cell-cycle exit at G0/G1. Furthermore, JOA significantly up-regulated the expression of CALML6 at both mRNA and protein level in MOLM-13 cells, and it remarkably down-regulated the expression of c-KIT at both transcription and protein levels in THP-1 cells. Taken together, we found that JOA is effective against MLLr AML cells by inducing cell differentiation. Moreover, MOLM-13 and MV4-11 cells are more sensitive to JOA than THP-1 cells.

It is revealed that martens tretinoin response up pathway was involved in the treatment of MOLM-13 cells with JOA (**Figure 6C** **)**. Genes including CALML6 (calcium binding protein calmodulin [CAM]), LPIN3, SEMA6B, FGR, LRFN3, SCIMP and TREM2 were enriched in this pathway, which regulates AML cell differentiation, RAR signaling, and epigenetic control (23). In this study, we showed that JOA had an anti-proliferative effect on MLLr AML cells by inducing cell differentiation. In addition, the expression of CALML6 were significantly up-regulated in MOLM-13 cells with JOA treatment. In addition, it is revealed that Ca^2+^/CaM activation is involved in dendritic cell-like differentiation of U937 cells (24). Therefore, it may be concluded that JOA up-regulated the expression of CALML6 which may be induce cell differentiation of MOLM-13 cells. The detailed mechanism need to be further investigated. Hence, these results indicate that the differentiation of MOLM-13 cells occurs perhaps *via* a martens tretinoin response up pathway activated by JOA.

Hematopoietic cell lineage is critical for the differentiation of basophils, eosinophils, macrophages, neutrophils, and myeloid-derived dendritic cells (25, 26). The lineage differentiation is down-regulated by c-KIT (CD117). c-KIT is a typical biomarker for undifferentiated hematopoietic cells and plays an important role in the differentiation and proliferation of leukemia cells (27, 28). Our present study showed that JOA treatment induced cell differentiation with morphological changes, increased the expression of CD11b and CD15, and alteration of cell adhesion molecules such as CD99, HLA-DRA, HLA-DQA1, HLA-DPA1, and HLA-DPB1 in THP-1 cells. JOA treatment also down-regulated the mRNA and protein level of c-KIT. Therefore, JOA-mediated differentiation of THP-1 cells might be through the alteration of lineage-specific target genes, and be related to hematopoietic cell lineage pathway *via* inhibition of c-KIT expression.

Cell adhesion molecules are a group of membrane glycoproteins and carbohydrate molecules that mediate cells to communicate with one another and their environment (29). They paly important role in cellular functions, including proliferation and differentiation. Here, JOA treatment resulted in the alteration of cell adhesion molecules such as CD11b and CD99 (less expression of them indicating the more differentiated leukemia cells) (30), CD15 and major histocompatibility complex (MHC) class II genes (immune regulation antigens including HLA-DRA, HLA-DQA1, HLA-DPA1, HLA-DPB1) in THP-1 cells. Hence, the alteration of cell adhesion molecules combined with the change of cell morphology and increasing expression of CD11b and CD15 indicates that the cell differentiation induced by JOA is associated with cell adhesion molecules pathway.

As describe above, JOA treatment increased expression of CD15 and CD11b, that are biomarkers of monocyte/macrophage and monocyte/granulocyte differentiation, respectively, suggesting that treatment with JOA promoted the commitment of THP-1 cells into the monocyte/macrophage or monocyte/granulocyte lineage. Moreover, JOA treatment altered the expression of genes including CD11b, HLA-DRA, HLA-DRB5, HLA-DQA1, HLA-DPA1, HLA-DPB1, NCF2, MARCO, TLR6, HLA-F, TUBB4A, FCAR, and TUBA8. These genes were enriched in the phagosome pathway. It was revealed that monocyte/macrophages are involved in phagocytosis in enteritis through the phagosome pathway (31, 32). Therefore, it suggested that JOA induces monocyte/macrophages differentiation of THP-1 cells, and activates the phagosome pathway. The result is in accordance with that phagosome pathway was commonly activated in both MV4-11 and Kasumi-1 AML cell lines treated with differentiation inducers, trichostatin A and 5-azacytidine (33).

Hence, the signaling pathways triggered in response to JOA treatment are different in MOLM-13 and THP-1 cells. It was shown that JOA mainly targeted the genes in the martens tretinoin response up pathway in the MOLM-13 cells, whereas it targeted the genes in cell adhesion molecules and hematopoietic cell lineage pathways in the THP-1 cells. These differences might be due to their different molecular characteristics (FLT3/ITD mutation vs wild -type FLT3).

In conclusion, our findings reveal that JOA possess anti-proliferation effect on MLLr AML cells perhaps through martens tretinoin response up pathway involving the CALML6 up-regulations, or hematopoietic cell lineage by depletion of c-KIT and cell adhesion molecules pathways depending on the cell type, which results in cell differentiation. JOA could overcome differentiation block in MLLr AML cells, suggesting that JOA could be a potential compound that merits further development to overcome differentiation blockade in MLLr AML patients.

## Data Availability Statement

The datasets presented in this study can be found in online repositories. The names of the repository/repositories and accession number(s) can be found here: GEO of NCBI, https://www.ncbi.nlm.nih.gov/geo/query/acc.cgi?acc=GSE167083 repository, accession number is GSE167083.

## Author Contributions

MQ: Conceptualization, methodology, and writing—original. YD: performed flow cytometry analysis. MZ: performed MTT assay. ZW: Data analysis. MJZ: performed Western blotting analysis. YZ: cell cycle analysis. HW: prepared the chemical reagent solutions. YK, YL, and H-ML: preparation of JOA. LW: Writing—review, editing, and supervision. ZH: Conceptualization and supervision. All authors contributed to the article and approved the submitted version.

## Funding

This work was supported by the National Natural Science Foundation of China (grants 8170016, 781370628 and 81570157) and Natural Science Foundation of Shandong Province (grant ZR2016HM47).

## Conflict of Interest

The authors declare that the research was conducted in the absence of any commercial or financial relationships that could be construed as a potential conflict of interest.
